# Isolated post-traumatic Intracholecystic Hemorrhage without Gallbladder Wall rupture: a rare diagnostic challenge

**DOI:** 10.1093/omcr/omag070

**Published:** 2026-05-10

**Authors:** Steffani Simone, Laudazi Mario, Chiocchi Marcello, Montella Viviana, Borrelli Stefano Pio, Garaci Francesco, Pignataro Marta, Mozzani Marcello, Manenti Guglielmo, Micillo Andrea

**Affiliations:** University of Rome Tor Vergata, Via Cracovia n.50, Rome, 00133, Italy; University of Rome Tor Vergata, Via Cracovia n.50, Rome, 00133, Italy; University of Rome Tor Vergata, Via Cracovia n.50, Rome, 00133, Italy; University of Rome Tor Vergata, Via Cracovia n.50, Rome, 00133, Italy; University of Rome Tor Vergata, Via Cracovia n.50, Rome, 00133, Italy; University of Rome Tor Vergata, Via Cracovia n.50, Rome, 00133, Italy; University of Rome Tor Vergata, Via Cracovia n.50, Rome, 00133, Italy; University of Rome Tor Vergata, Via Cracovia n.50, Rome, 00133, Italy; University of Rome Tor Vergata, Via Cracovia n.50, Rome, 00133, Italy; Poligest SpA Casa di Cura Villa Delle Querce, Radiology Department, Italy

**Keywords:** blunt abdominal trauma, Intracholecystic hemorrhage, multiphasic CT, active extravasation, laparoscopic cholecystectomy

## Abstract

Blunt abdominal trauma rarely results in isolated gallbladder injury. Intracholecystic hemorrhage without concomitant liver injury or gallbladder wall rupture is an uncommon presentation. We report the case of a 55-year-old male presenting with epigastric pain following thoracoabdominal trauma. While initial non-contrast CT showed a distended gallbladder with some hyperdense endoluminal fluid, subsequent contrast-enhanced CT revealed active contrast extravasation into the gallbladder lumen, diagnostic of active bleeding, in the absence of hepatic injury. Despite initial hemodynamic stability prompting conservative management, follow-up imaging demonstrated a gallbladder lumen completely filled with hyperdense material and mild thickening of the gallbladder walls. This finding was interpreted as an increase in intracholecystic blood content, likely superimposed on the concomitant biliary excretion of contrast medium from the initial scan, necessitating laparoscopic cholecystectomy. This case highlights the role of multiphasic CT in diagnosing isolated gallbladder hemorrhage, which may require surgery despite intact walls.

## Learning Points

- Isolated intracholecystic hemorrhage is a rare consequence of blunt trauma and can occur without liver injury or gallbladder rupture.
- Intracholecystic hyperdensities should not be consistently assumed to be sludge; in the context of trauma, they indicate hemorrhage until proven otherwise.
- Multiphasic CT is essential; active contrast extravasation is the hallmark of ongoing bleeding.
- Even in hemodynamically stable patients, conservative management requires close radiological monitoring, as progressive distension may mandate surgical intervention.

## Introduction

Blunt abdominal trauma encompasses a wide spectrum of injuries, most commonly affecting solid organs such as the spleen and liver [1]. Injuries to the gallbladder are relatively infrequent, occurring in approximately 1%–2% of patients sustaining significant blunt abdominal trauma, and are typically associated with damage to adjacent structures [2]. Isolated intracholecystic hemorrhage—bleeding confined within the gallbladder lumen secondary to trauma without wall rupture or associated liver injury—is an extremely rare entity; Wiebe et al. noted fewer than 10 cases in the literature [3]. We present a case of isolated post-traumatic intracholecystic hemorrhage diagnosed via multiphasic CT, emphasizing the diagnostic nuances and types of management from conservative to surgical intervention.

## Case presentation

**Figure 1:**
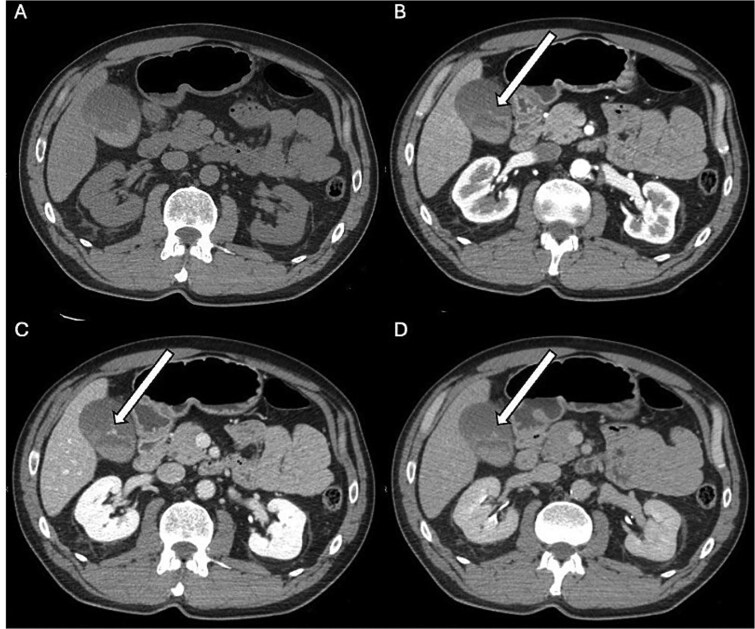
Initial multiphasic CT scan. (A) Non-contrast axial image showing the gallbladder containing spontaneously hyperdense material, consistent with blood. (B) Arterial phase, (C) venous phase, and (D) delayed phase. In these contrast-enhanced phases, focal contrast extravasation ("blush") is observed pooling within the lumen of the gallbladder body (arrow); this finding, best appreciated in the venous phase (C), is consistent with active bleeding.

**Figure 2:**
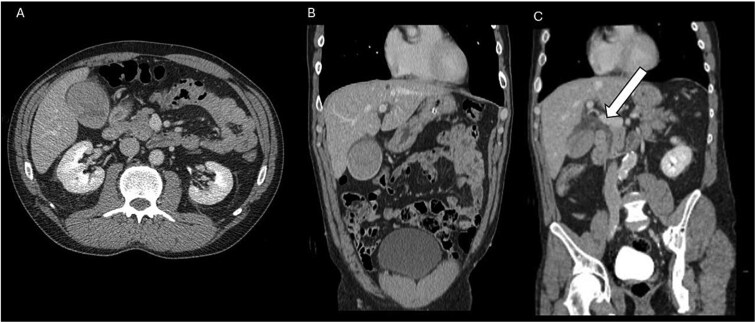
Follow-up CT scan. (A) Axial image showing the gallbladder lumen completely filled with hyperdense material. This finding is consistent with extensive endoluminal hemorrhage, likely compounded by the concurrent vicarious excretion of contrast medium from the previous administration, accompanied by the new appearance of pericholecystic fluid and mild thickening of the gallbladder walls. (B) Coronal reconstruction in the venous phase confirming the findings. (C) Coronal reconstruction in the venous phase revealing, in addition to the described findings, the new appearance of extrahepatic biliary tract dilation (arrow).

A 55-year-old male presented to the emergency department complaining of acute, moderate, non-radiating pain localized to the epigastrium and right hypochondrium. The symptoms onset followed a blunt trauma to the right lower chest and upper abdomen sustained during a mechanical fall. The patient had no significant past medical history and denied the use of anticoagulants or antiplatelet agents. Upon admission, the patient was hemodynamically stable (BP 135/85 mmHg, HR 88 bpm). Admission laboratory results were unremarkable with a hemoglobin level of 15,9 g/dl. Due to the mechanism of injury and localized tenderness, a CT scan of the abdomen and pelvis was performed. The initial non-contrast phase revealed a hydropic gallbladder with smooth, intact walls and no pericholecystic fluid. However, the lumen was partially filled with high-attenuation material, raising suspicion for intracholecystic hemorrhage (Fig. 1A). The intraluminal blood content demonstrated a density value of 71 ± 10 Hounsfield Units (mean ± SD) on the basal scan; notably, while biliary sludge typically appears hyperdense, it generally exhibits lower density values. No injuries were observed in the liver, spleen, pancreas, or kidneys. To investigate further, a contrast-enhanced study was completed. The examination was acquired using a triphasic protocol with the intravenous administration of 110 mL of iodinated contrast medium (Iomeprol 350 mg/ml) at a flow rate of 4 mL/s. This demonstrated active extravasation of contrast material pooling within the gallbladder lumen, confirming active bleeding (Fig. 1B–D). Given the patient's hemodynamic stability, absence of peritonitis, and the containment of the bleeding with normal level of hemoglobin, a conservative management strategy was initially adopted. However, a follow-up contrast-enhanced CT performed 12 hours later showed the lumen of the gallbladder completely filled with hyperdense material. This finding is consistent with the accumulation of blood clots, likely accentuated by the concomitant biliary excretion of contrast medium from the recent prior administration. Additionally, new pericholecystic fluid and mild mural thickening of the gallbladder were noted (Fig. 2). Concurrently, laboratory tests showed a minimal decrease in hemoglobin to 14.8 g/dl and then to 13.6 g/dl in two days, which remained within the range. Follow-up labs showed CRP 64.4 mg/l, ALT 80 U/l, GGT 155 U/l, total bilirubin 1.58 mg/dl. The radiological evolution was suggestive of developing inflammatory changes and raised concerns regarding potential complications. Consequently, the patient underwent a laparoscopic cholecystectomy. The procedure was uncomplicated, and histopathological examination confirmed extensive luminal hemorrhage with blood clots and mild acute inflammatory changes. Following the removal of the gallbladder, macroscopic assessment also confirmed that the wall appeared intact. The patient was discharged on the fourth postoperative day in good condition.

## Discussion

This case illustrates an exceedingly rare complication of blunt abdominal trauma: isolated intracholecystic hemorrhage with an intact gallbladder wall and preserved liver parenchyma [2, 3]. The mechanism likely involves shearing forces from rapid deceleration or direct impact, causing the tearing of small intramural vessels or branches of the cystic artery, sparing the structural integrity of the wall itself [4, 5]. CT imaging is paramount for diagnosis. While non-contrast CT is sensitive for high-attenuation blood products (hemobilia), differentiating this from biliary sludge can be challenging [6]. The definitive sign, as seen in this case, is active contrast extravasation ("blush") within the lumen on multiphasic CT [7, 8]. This finding distinguishes active hemorrhage from prior hematoma and guides the urgency of management [7, 8].

It is crucial to emphasize that not all hyperdense material observed within the gallbladder on non-contrast CT should be automatically labeled as biliary sludge [8]. Although sludge is a common finding, the differential diagnosis for intracholecystic hyperdensities is broad and includes radio-opaque gallstones, milk of calcium bile, vicarious excretion of intravascular contrast material, and hemorrhage [8]. In this context, the patient's anamnesis and clinical presentation are indispensable tools for guiding radiological interpretation [8]. While a history of biliary colic points towards lithiasis, a history of recent trauma mandates considering hemorrhage as the primary diagnosis until proven otherwise [8]. Solely relying on density values without integrating the clinical scenario can lead to misdiagnosing life-threatening hemorrhage as benign sludge [8, 9].

Management strategies for traumatic gallbladder injury typically depend on hemodynamic status and the extent of injury [9]. While cholecystectomy is standard for avulsion or perforation, contained hemorrhage offers a potential window for conservative management or endovascular embolization [9]. However, endovascular embolization is often avoided due to the gallbladder’s terminal blood supply, where cystic artery occlusion carries a significant risk of ischemic cholecystitis [4, 9]. In this case, conservative management failed as evidenced by increasing distension and pericholecystic fluid on follow-up imaging, indicating that the bleeding, although contained, was significant enough to threaten organ viability [1, 10]. Therefore, early cholecystectomy remains the definitive treatment to prevent complications such as delayed rupture or cholecystitis [1, 10].

Conclusion: Isolated intracholecystic hemorrhage without wall rupture is a rare post-traumatic entity. Multiphasic CT is essential for diagnosis and follow-up, identifying active contrast extravasation to distinguish hemorrhage from biliary sludge. Even in hemodynamically stable patients, radiological evidence of progressive gallbladder distension indicates significant bleeding and necessitates laparoscopic cholecystectomy to prevent delayed rupture.
